# Lipocalin-2 in Diabetic Complications of the Nervous System: Physiology, Pathology, and Beyond

**DOI:** 10.3389/fphys.2021.638112

**Published:** 2021-02-05

**Authors:** Anup Bhusal, Won-Ha Lee, Kyoungho Suk

**Affiliations:** ^1^Department of Pharmacology, School of Medicine, Kyungpook National University, Daegu, South Korea; ^2^BK21 Plus KNU Biomedical Convergence Program, Department of Biomedical Science, School of Medicine, Kyungpook National University, Daegu, South Korea; ^3^School of Life Sciences, BK21 FOUR KNU Creative BioResearch Group, Kyungpook National University, Daegu, South Korea; ^4^Brain Science and Engineering Institute, Kyungpook National University, Daegu, South Korea

**Keywords:** lipocalin-2, immunity, energy metabolism, diabetes, neuroinflammation, diabetic complications, nervous system

## Abstract

Lipocalin-2 (LCN2) is a 25 kDa secreted protein that belongs to the family of lipocalins, a group of transporters of small hydrophobic molecules such as iron, fatty acids, steroids, and lipopolysaccharide in circulation. LCN2 was previously found to be involved in iron delivery, pointing toward a potential role for LCN2 in immunity. This idea was further validated when LCN2 was found to limit bacterial growth during infections in mice by sequestering iron-laden siderophores. Recently, LCN2 was also identified as a critical regulator of energy metabolism, glucose and lipid homeostasis, and insulin function. Furthermore, studies using *Lcn2* knockout mice suggest an important role for LCN2 in several biobehavioral responses, including cognition, emotion, anxiety, and feeding behavior. Owing to its expression and influence on multiple metabolic and neurological functions, there has emerged a great deal of interest in the study of relationships between LCN2 and neurometabolic complications. Thorough investigation has demonstrated that LCN2 is involved in several neurodegenerative diseases, while more recent studies have shown that LCN2 is also instrumental for the progression of diabetic complications like encephalopathy and peripheral neuropathy. Preliminary findings have shown that LCN2 is also a promising drug target and diagnostic marker for the treatment of neuropathic complications from diabetes. In particular, future translational research related to LCN2, such as the development of small-molecule inhibitors or neutralizing antibodies against LCN2, appears essential for exploring its potential as a therapeutic target.

## Introduction

Diabetes is a syndrome defined by the presence of abnormally high blood glucose levels or hyperglycemia (Guthrie and Guthrie, 2004; Skyler et al., 2017). Hyperglycemia is known to cause a wide variety of metabolic disturbances and affect both the peripheral nervous system (PNS) and central nervous system (CNS), either directly or indirectly, which can lead to several complications collectively referred to as diabetic neuropathy over a long period of time (Sima et al., 2004; Malone, 2016). Diabetes mainly affects nerves in hands, feet, legs, and arms and is long considered a disease of the PNS. However, there is now increasing evidence of diabetic effects on the CNS as part of a condition called diabetic encephalopathy (Selvarajah et al., 2011). The pathogenesis of diabetic encephalopathy has not been fully defined, yet appears to show similarities to the progression of diabetic peripheral neuropathy (DPN) (Manschot et al., 2008).

Recently, there has been wide agreement that excessive glial activation is a key mechanism in both CNS‐ and PNS-related complications of diabetes involving the release of proinflammatory cytokines. Glial cells play an essential role in maintaining the normal function of nervous tissues. In the PNS, neurons are intimately associated with numerous glial cells; the neuronal soma is enclosed by satellite glial cells (SGCs) and axons are covered along their length by Schwann cells (Goncalves et al., 2018). Similarly, neurons are in close contact with astrocytes, microglia, oligodendrocytes, and Müller glial cells in the CNS. These glial cells are responsible for the uptake and metabolism of glucose in the nervous system. Fluctuations in glucose levels activate them, causing activation of MAPK/PI3K/Akt/NF-κB signaling and release of proinflammatory factors (Quincozes-Santos et al., 2017; Hsieh et al., 2019). This inflammatory activation of glial cells triggers further metabolic deterioration and affects both small and large nerve fibers, resulting in nerve dysfunction that is characteristic of diabetic neuropathy (Goldberg, 2009; Pop-Busui et al., 2016).

Lipocalin-2 (LCN2), also known as neutrophil gelatinase-associated lipocalin (NGAL), siderocalin, and 24p3, is a member of the lipocalin superfamily and is a pleiotropic mediator of various physiological and pathological processes (Xiao et al., 2017; Bhusal et al., 2019b). LCN2 so far is known to act through two major membrane-bound receptors: megalin, also known as low-density lipoprotein-related protein 2 (LRP2), and 24p3R, also referred to as solute carrier SLC22A17 or brain-type organic cation transporter (BOCT). LCN2 functions through these receptors in an iron-dependent manner, where apo-LCN2 chelates iron inside the cell, releases to the extracellular medium, reduces intracellular iron concentration, and finally causes cellular apoptosis. On the other hand, holo-LCN2 increases intracellular iron concentration and prevents cellular apoptosis by decreasing the expression of the proapoptotic protein Bcl-2-like protein 11 (Bim) (Devireddy et al., 2005). However, this hypothesis has lately been challenged, as HeLa cells expressing BOCT receptors did not exhibit cellular iron efflux following LCN2 treatment. Moreover, LCN2, even at higher doses, did not induce apoptosis in hematopoietic cell line (Correnti et al., 2012). Studies also failed to show the interaction between LCN2 and BOCT (Bennett et al., 2011; Correnti et al., 2012). Similarly, conflicting views have also been reported regarding the role of LCN2 in several metabolic conditions, as reviewed earlier by our group (Bhusal et al., 2019b). In light of these findings, LCN2 has recently been proposed to play an important role in the development of diabetic complications. Here, we review the physiological as well as pathological role of LCN2 in the nervous system, and discuss the latest reports as to how it could be used as a target for the treatment of diabetic neurological complications. In this review, the term “diabetic neuropathy” is used throughout the manuscript in reference to a disease of the peripheral and central nervous system, unless otherwise specified.

## The Role of LCN2 in Nervous System Physiology and Pathology

Lipocalin-2 is produced by mammalian hosts to bind bacterial siderophores and sequester free iron as part of innate immune defenses against bacterial infection; however, thus far, its role in the nervous system is less well understood. The upregulation of LCN2 in the brain was first observed in response to peripheral turpentine-induced inflammation (Liu and Nilsen-Hamilton, 1995). In addition, the study of LCN2 function in various neuroinflammatory conditions largely began with the observation of LCN2 expression in microglia (Lee et al., 2007) and astrocytes (Lee et al., 2009) in the CNS. However, the physiological role of LCN2 has not been studied due to its low or undetectable expression in healthy adult brains (Ip et al., 2011; Chakraborty et al., 2012; Kim et al., 2017; Kang et al., 2018) or peripheral nerves (Jeon et al., 2013; Bhusal et al., 2020), although some studies have demonstrated LCN2 protein expression in the hippocampus (Mucha et al., 2011; Chia et al., 2015; Furukawa et al., 2017), cortex, and amygdala (Furukawa et al., 2017) of normal rodents. Furthermore, strong constitutive expression of the LCN2 receptor 24p3R in the brain of normal mice has been reported (Ip et al., 2011; Chia et al., 2015). It is, therefore, possible that basal expression of LCN2 in different regions of the brain may help in the defense of the CNS against pathogens. Beyond resisting infections, high levels of LCN2 in other regions of the brain may contribute to iron transport in these regions under normal conditions.

### LCN2 in Stress, Anxiety, Depression, and Cognitive Function

Recent studies using *Lcn2* knockout (KO) animals have improved understanding of the role of LCN2 in the regulation of physiological conditions like stress, emotion, and memory. In one such study, *Lcn2* KO mice displayed increased anxiety and depressive-like behaviors and mild spatial reference memory impairments (Ferreira et al., 2013). These altered phenotypes were associated with hyperactivation of the hypothalamic-pituitary-adrenal axis, reflected in the increased levels of corticosteroids at both the morning and night periods in the *Lcn2*-deficient mice. Furthermore, the hippocampal neuronal morphology of *Lcn2* KO mice displayed hypertrophy of granular and pyramidal neurons at the ventral hippocampus, a region implicated in emotional behavior, as well as neuronal atrophy at the dorsal hippocampus, a region implicated in memory and cognition (Ferreira et al., 2013). Another study by the same group found that *Lcn2* deficiencies lead to higher proportion of progenitor cells in hippocampus exiting the cell cycle and progressing toward apoptotic cell death (Ferreira et al., 2018). Furthermore, deletion of *Lcn2* in neural stem cells induced endogenous oxidative stress, cell cycle arrest, and cell death in an iron-mediated manner (Ferreira et al., 2018). In a later extension of their study, the impaired hippocampal neurogenesis observed in *Lcn2* KO mice was relieved by voluntary running, which counteracted oxidative stress and promoted cell cycling of neural stem cells, resulting in the partial reduction of anxiety and improved contextual behavior (Ferreira et al., 2019). In line with this, the ablation of *Lcn2* gene proved deleterious and promoted a stress-induced increase in spine density, which correlated with higher excitability of CA1 neurons and stress-induced anxiety (Mucha et al., 2011).

### LCN2 in Food Intake Regulation

Mosialou et al. (2017) recently revealed an unexpected role of LCN2 regarding feeding behavior of mice. In that study, LCN2 secreted from bone crosses the blood-brain barrier, binds to melanocortin 4 receptors (MC4R) in neurons of the hypothalamus, and activates an MC4R-dependent anorexigenic pathway (Mosialou et al., 2017). This finding was extended to baboon, macaque, and human, where LCN2 acted as a satiety factor, and failure to stimulate postprandial LCN2 in individuals with obesity contributed to metabolic dysregulation (Petropoulou et al., 2020).

However, these observations should be carefully considered. In the study by Guo et al. (2010), indirect calorimetry measurements revealed no difference in food intake behavior between wild-type and *Lcn2* KO mice. In another study, *Lcn2*-deficient mice displayed no alteration in food intake upon Celastrol treatment, which is known to increase LCN2 levels in hypothalamus (Feng et al., 2019). Recently, LCN2-overexpressing transgenic mice showed an increased food intake (Principi et al., 2019). Similarly, food intake was increased when mice were injected with LCN2 protein (Paton et al., 2013). These controversial findings warrant further investigation into the mechanisms how LCN2 regulates food intake behavior.

### LCN2 in Inflammatory and Other Neurological Disorders

Beyond the physiological roles of LCN2, recent studies have shown an increase in expression of and an important role for LCN2 in various pathological states. LCN2 has been found to regulate diverse cellular processes and phenotypes in the nervous system, including cell death and survival (Lee et al., 2007; Naude et al., 2012; Bi et al., 2013), cell migration and morphology (Lee et al., 2011, 2012; Rathore et al., 2011), and the functional polarization of microglia (Jang et al., 2013b; McPherson et al., 2014) and astrocytes (Jang et al., 2013a). These functional characteristics of LCN2 have been exploited by many researchers to study the role of LCN2 in different neurological disorders, including neuroinflammation (Lee et al., 2011; Jin et al., 2014a, 2018; Mondal et al., 2020), Alzheimer’s disease (Naude et al., 2012; Dekens et al., 2018; Eruysal et al., 2019), ischemic stroke (Jin et al., 2014b; Xing et al., 2014; Hochmeister et al., 2016; Ranjbar Taklimie et al., 2019; Zhao et al., 2019), experimental autoimmune encephalomyelitis (Berard et al., 2012; Marques et al., 2012; Nam et al., 2014), brain and spinal cord injuries (Chia et al., 2011; Rathore et al., 2011; Dong et al., 2013), malignant gliomas (Suk, 2012), and pain hypersensitivity (Poh et al., 2012; Jeon et al., 2013; Jha et al., 2013, 2014). As a result of its active participation in the pathogenesis of various neurological diseases, LCN2 can be considered a promising therapeutic target for both prognostic and diagnostic purposes. Recently, the number of studies describing a role for LCN2 in metabolic homeostasis (Yan et al., 2007; Jun et al., 2011; Guo et al., 2012, 2016; Mosialou et al., 2020) and the pathogenesis of diabetes-related complications is on the rise, indicating similar importance of LCN2 in diabetes-related neurological disorders.

## LCN2 in Neurological Complications of Diabetes

Recently, several clinical studies have demonstrated a close relationship between LCN2 expression and the risk of impaired glucose metabolism (Wang et al., 2007; Yang et al., 2009; Huang et al., 2012). Furthermore, LCN2 levels have been linked to diabetic complications like retinopathy (Chung et al., 2016; Wang et al., 2020) and nephropathy (Wu et al., 2014; Papadopoulou-Marketou et al., 2017; Sisman et al., 2020). However, the precise mechanisms underlying the role of LCN2 in diabetic complications remain unclear and several plausible explanations have been suggested. LCN2 has been reported to deliver iron to cells, causing intracellular iron overload and results in oxidative stress, cellular degeneration, and increased levels of advanced glycation end-product (AGE) receptors for AGE binding (Ciudin et al., 2010). LCN2 has been shown to activate metalloproteinase-9 (MMP-9) by forming a stable complex with MMP-9 (Yan et al., 2001). MMP-9 activation may then facilitate an increase in vascular permeability through the proteolytic degradation of tight junction proteins (Opdenakker and Abu El-Asrar, 2019). LCN2 may also be linked to the production of AGE (Chung et al., 2013; Petrica et al., 2015), which interacts with plasma membrane-localized receptors for AGEs (RAGE) to alter intracellular signaling, gene expression, and the release of proinflammatory molecules and free radicals (Singh et al., 2014). In addition, LCN2 is involved in immune reactions and inflammatory processes (Lee et al., 2011; Jin et al., 2014a, 2018; Mondal et al., 2020). These mechanisms involving LCN2 are all relevant in the nervous system, as LCN2 is significantly expressed in both CNS and PNS following the onset of diabetes, making LCN2 a potential focus in the study of diabetic neuropathy.

### LCN2 in Diabetic Encephalopathy

**Figure 1:**
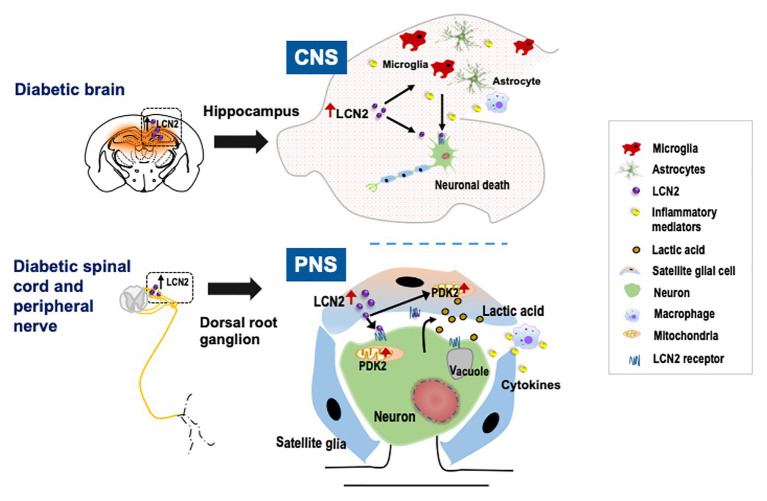
Schematic representation of the role of lipocalin-2 (LCN2) in diabetic neuropathy. Diabetes leads to the upregulation of LCN2 in the hippocampus of the brain and in satellite glial cells (SGCs) of the dorsal root ganglion (DRG). In the central nervous system (CNS), induction of LCN2 causes activation of glial cells, macrophage infiltration, and upregulation of inflammatory cytokines leading to diabetic encephalopathy. In the peripheral nervous system (PNS), it has been suggested that LCN2 released from the SGCs in the DRG acts on SGCs as well as nearby neurons to induce pyruvate dehydrogenase kinase-2 (PDK2) expression. The LCN2-PDK2 axis-mediated glycolytic metabolic shift in the DRG results in the production of lactic acid, which causes an acidic microenvironment that eventually causes neuronal damage, leading to diabetic peripheral neuropathy.

Diabetic encephalopathy is a chronic complication of diabetes mellitus characterized by oxidative stress, impaired microvascular permeability, neurogenesis, cognitive functions, and electrophysiological, neurochemical, and structural abnormalities (Cai et al., 2011; Ho et al., 2013; Chen et al., 2018). People with diabetes mellitus have increased levels of proinflammatory cytokines such as C-reactive protein, TNF-α, and IL-6 (Esposito et al., 2002; Tangvarasittichai et al., 2016). Recently, a study reported the upregulation and pathological role of LCN2 in the hippocampus of an insulin-deficient diabetes model created by streptozotocin injection (Bhusal et al., 2019a). In this study, deletion of the *Lcn2* gene ameliorated diabetes-induced reactive gliosis and expression of proinflammatory cytokines in the hippocampus of diabetic mice. Moreover, *Lcn2* KO diabetic mice showed decreased neuronal loss in the hippocampus compared to wild-type diabetic animals, an effect correlated with improved cognitive behavior (Bhusal et al., 2019a) (Figure 1). Previously, the same group reported increased plasma levels in patients with mild cognitive impairments (Choi et al., 2011), supporting the correlation between LCN2 and cognitive deficits. Another study conducted using four independent cohorts with a large number of samples concluded that cerebrospinal fluid LCN2 is a promising biochemical marker for the differential diagnosis of neurodegenerative dementias (Llorens et al., 2020). These findings indicate that LCN2 has significant translational potential for several brain-related complications of diabetes.

Recently, a study reported increased serum and hippocampal LCN2 levels in ob/ob mice. In this study, ob/ob mice showed impaired spatial learning behavior, which was improved following a calorie-restricted diet that correlated with decreased expression of LCN2 in the hippocampus (Park et al., 2019). Another study suggested that increasing LCN2-mediated iron uptake may be required for Toll-like receptor 4/endosome-related WD repeat and FYVE domain-containing 1 (Wdfy1)-signaling during hippocampal neuroinflammation in ob/ob mice (Jin et al., 2020). Furthermore, a study using a combination of high-fat diet and fructose showed that elevated hippocampal and peripheral LCN2 levels mediate the impact of chronic inflammation on the CNS, which is associated with behavioral dysfunction (de Sousa Rodrigues et al., 2017). Additionally, recent studies attempted to evaluate the role of a chronic high-fat diet in neuroinflammation with respect to myeloid sirtuin1 (SIRT1) and LCN2 function. It was found that SIRT1 promotes hippocampal inflammation in association with LCN2 levels (Kim et al., 2018; Park et al., 2019).

Obesity and high-fat diet are associated with an increased risk of developing insulin resistance and type 2 diabetes. Considering evidence that circulating LCN2 levels are associated with hyperglycemia, insulin resistance, and metabolic syndrome based on *in vitro* (Yan et al., 2007; Chan et al., 2016), *in vivo* (Wang et al., 2007; Guo et al., 2010), and clinical studies (Cakal et al., 2011), it is speculated that LCN2 may be involved in the regulation of insulin sensitivity in the brain, although the relationship between LCN2 expression and insulin resistance in the brain has not been investigated. Taken together, specific mechanisms relating to the association between LCN2 and IR in the brain can be additional factors aggravating diabetic encephalopathy.

### LCN2 in Diabetic Peripheral Neuropathy

Diabetic peripheral neuropathy is the most common microvascular complication of diabetes mellitus characterized by inflammation, oxidative stress, and mitochondrial dysfunction (Roman-Pintos et al., 2016). Based on the previous observations of the induction of LCN2 expression and its role in neuroinflammation in the brains of mice with diabetes or various demyelinating diseases (Nam et al., 2014; Chun et al., 2015; Al Nimer et al., 2016), Bhusal et al. (2020) have recently provided evidence for a potentially novel role of LCN2 in the progression of DPN. They found that LCN2 is expressed by SGCs in the dorsal root ganglion (DRG) and by Schwann cells in the sciatic nerves of diabetic mice. This LCN2 expression was dependent on high glucose levels, as evidenced by the decrease in LCN2 levels in the DRG and improvement in nerve conduction velocity in diabetic mice following insulin treatment. Furthermore, their study using *Lcn2* KO mice showed a decrease in inflammation in the DRG and sciatic nerve and reduction in the consequent DPN phenotype; however, the mechanisms through which *Lcn2* deficiency attenuates DPN remain unclear.

Pyruvate dehydrogenase kinase-2 (PDK2), a mitochondrial enzyme, is known to drive a metabolic shift in dorsal root ganglia, which induces neuroinflammation, lactic acid build-up and ultimately produces painful DPN (Rahman et al., 2016). Similarly, multiple studies have reported that LCN2 increases mitochondrial reactive oxygen species, alters mitochondrial oxidative phosphorylation, and impairs overall mitochondrial activity (Yang et al., 2012; Song et al., 2018; Chella Krishnan et al., 2019). Given the proven role of LCN2 and PDK2 in mitochondrial activity, Bhusal et al. (2020) investigated whether there is any functional relationship between LCN2 and PDK2 in terms of neuroinflammation and lactic acid production in DPN. In their study, PDK2 overexpression using adenoviruses in the DRG in *Lcn2* KO mice potentiated inflammation and DPN, which was not observed when LCN2 was overexpressed in *Pdk2* KO mice. Furthermore, LCN2 increased expression of PDK2 in SGC culture, which was shown using pharmacological blockade to be dependent on peroxisome proliferator-activated receptors. From these findings, the authors concluded that LCN2 acts as an upstream regulator of PDK2 in the SGCs of DRG, which potentiates neuroinflammation, lactate surge, and consequent DPN (Bhusal et al., 2020) (Figure 1). These findings ultimately put forward a novel mechanism for an LCN2-PDK2-lactic acid axis in diabetes-induced neuroinflammation and consequent diabetic complications like neuropathy.

## Concluding Remarks

During the past few decades, there has been an increased understanding of the relationships among metabolic syndrome, adipokines, and inflammatory diseases. In this regard, LCN2 can be considered one of the mediators responsible for inflammation in complications associated with diabetes. Recent studies have provided essential evidence of LCN2 as a major player linked to metabolism, inflammation, and neuropathy. LCN2 expression may be a useful early diagnostic biomarker for diabetic neuropathy; however, further validation using human samples derived from larger multi-institutional cohorts is needed. Further research into the function of LCN2 will guide our understanding of its potential use as a diagnostic and therapeutic agent and will create new opportunities for improving the care of patients with diabetic neuropathy.

## Author Contributions

AB, W-HL, and KS wrote the manuscript. All authors contributed to the article and approved the submitted version.

### Conflict of Interest

The authors declare that the research was conducted in the absence of any commercial or financial relationships that could be construed as a potential conflict of interest.
